# Supplementary material for: Content-rich biological network constructed by mining PubMed abstracts
Source: BMC Bioinformatics. 2004 Oct 8;5:147. doi: 10.1186/1471-2105-5-147 (PMC528731; doi:10.1186/1471-2105-5-147)
Supplement: Additional File 2 — The original results of the above study (non-essential files are deleted to keep the file size under the limit set by BMC bioinformatics). [file 1471-2105-5-147-S2.bz2 › chilibotAdditionalFile2/dip20/373ID7680645E8296/html/FGF1_FGFR4.html]

 


 **FGF1** and **FGFR4** 
  
Found 127 abstracts in PubMed, retrieved 20.  
 

 What does Google say? 
 PDF only 
| .edu only 

---

**Interactive relationship** (e.g. stimulation, inhibition, etc)

**Inhibitory relationship**- FGF  [ **FGF1** ]  19 also stimulated MAPK activation, an effect that was abrogated by a soluble dominant negative dn form of  **FGFR4** .  Ref: 12169442 Am J Physiol Endocrinol Metab, 2002
- We show that inhibition of  **FGFR4**  signal results in an arrest of muscle progenitor differentiation, which can be rapidly reverted by the addition of exogenous FGF  [ **FGF1** ] , rather than a modification in their proliferative capacities.  Ref: 12223412 Development, 2002
**Neutral relationship**- We show here that oligosaccharides shorter than 8 sugar units support activation of FGFR2 IIIb by FGF 1  [ **FGF1** ]  and interaction of  **FGFR4**  with FGF 1  [ **FGF1** ] .  Ref: 11714710 J Biol Chem, 2002
- We conclude that  **FGFR4**  mediates FGF  [ **FGF1** ]  19 signaling to the PRL promoter.  Ref: 12169442 Am J Physiol Endocrinol Metab, 2002
- We show that FGF  [ **FGF1** ]  19, which selectively binds  **FGFR4** , can induce prolactin PRL but not growth hormone expression.  Ref: 12169442 Am J Physiol Endocrinol Metab, 2002
- **FGF1** , FGF7, FGF9, FGF10, FGF11, and FGF18 were also expressed in the pancreas at varying time points and levels, as well as FGF  [ **FGF1** ]  receptor forms FGFR1b, FGFR1c, FGFR2b, FGFR2c, FGFR3b, and  **FGFR4** .  Ref: 12666204 Dev Dyn, 2003

**Non-interactive relationship** (e.g. studied together, co-existance, homology, etc.)

- These findings represent the first description of an endogenous soluble C terminally truncated  **FGFR4**  isoform with FGF  [ **FGF1** ]  modulatory properties.  Ref: 11549253 Biochem Biophys Res Commun, 2001
- In contrast, Ets enhanced FGF  [ **FGF1** ]  19 induced PRL activation but failed to confer an effect for FGF  [ **FGF1** ]  19 on the  **FGFR4**  promoter.  Ref: 12169442 Am J Physiol Endocrinol Metab, 2002
- Formalin fixed, paraffin embedded normal tissues and 60 well characterized GEP endocrine tumors were immunostained using specific antibodies directed against various GEP hormones, aFGF  [ **FGF1** ] , FGFR1, FGFR2, FGFR3, and  **FGFR4** .  Ref: 11759058 Appl Immunohistochem Mol Morphol, 2001
- The authors focused on FGFR 4  [ **FGFR4** ]  expression in astrocytoma because the FGF  [ **FGF1** ]  expression increases as the tumor malignancy progresses.  Ref: 11958417 Neurol Res, 2002
- This provides an initial description of the expression in the developing skull of the genes encoding the secreted molecules BMP 2, BMP 4, BMP 7, the transmembrane FGF  [ **FGF1** ]  receptors FGFR 1, FGFR 2, FGFR 4  [ **FGFR4** ] , the transcription factors Msx1, Msx2, and Twist, as well as Goosecoid and the early membranous bone differentiation marker osteopontin.  Ref: 12710966 Dev Biol, 2003
- We demonstrate that FXR directly regulates expression of fibroblast growth factor 19 FGF  [ **FGF1** ]  19, a secreted growth factor that signals through the  **FGFR4**  cell surface receptor tyrosine kinase.  Ref: 12815072 Genes Dev< MedlineTA>Genes Dev, 2003
- In chick embryos, most if not all, replicating myoblasts present within the skeletal muscle masses express high levels of the FGF  [ **FGF1** ]  receptor FREK  **FGFR4** , suggesting an important role for this molecule during myogenesis.  Ref: 12223412 Development, 2002
- Of the four fibroblast growth factor FGF  [ **FGF1** ]  receptor genes expressed in adult liver,  **FGFR4**  is expressed specifically in mature hepatocytes.  Ref: 12466116 Am J Pathol, 2002
- This soluble  **FGFR4**  isoform sFGFR4 abrogates the effect of FGF 1  [ **FGF1** ]  induced MAPK phosphorylation and PRL gene activation.  Ref: 11549253 Biochem Biophys Res Commun, 2001
- We report the identification of a novel pituitary tumor derived, N terminally truncated isoform of FGF  [ **FGF1** ]  receptor 4 ptd  **FGFR4** .  Ref: 11781352 J Clin Invest, 2002
- Thus, we examined whether FGF  [ **FGF1** ]  19 might regulate its own receptor through the Ets Ik element in the  **FGFR4**  promoter.  Ref: 12169442 Am J Physiol Endocrinol Metab, 2002
